# NS-segment-based reporter influenza A viruses: engineering strategy, applications, and limitations

**DOI:** 10.1128/jvi.01099-26

**Published:** 2026-08-26

**Authors:** Ramya S. Barre, Himadri Nath, Aitor Nogales, Elsayed M. Abdelwhab, Ahmed M. Elsayed, Luis Martinez-Sobrido

**Affiliations:** 1 Texas Biomedical Research Institute 7075, San Antonio, Texas, USA; 2 Department of Microbiology, Immunology, and Molecular Genetics, University of Texas Health Sciences Center at San Antonio, San Antonio, Texas, USA; 3 Center for Animal Health Research, CISA-INIA-CSIC 54402 , Madrid, Spain; 4 Institute of Molecular Virology and Cell Biology, Friedrich-Loeffler-Institut, Isle of Riems, Greifswald, Germany; 5 Center of Scientific Excellence for Influenza Viruses, National Research Centre, Giza, Egypt; New York University Department of Microbiology, New York, New York, USA

**Keywords:** reporter gene, IAV, luciferase, Venus, antiviral bioassay, viral infection

## Abstract

Reporter-expressing recombinant influenza A viruses (IAVs) have emerged as powerful tools for studying viral infection, host–pathogen interactions, and the identification and characterization of prophylactic and/or therapeutic countermeasures for the treatment of IAV infections. By incorporating a reporter gene, such as fluorescent, recombinase, or luciferase proteins, into the viral genome, these genetically engineered recombinant IAVs enable real-time visualization and quantification of infection *in vitro*, *ex vivo*, and *in vivo* to detect the presence of IAV in infected cells or animal models of infection. Among the various approaches to express reporter genes from the viral genome, fusion of fluorescent or luciferase proteins to the C-terminus of the non-structural 1 (NS1) protein has been successfully used to generate replication-competent reporter-expressing IAVs. This minireview summarizes the strategies used to generate IAV-expressing reporter genes, particularly from the non-structural (NS) viral segment 8, and their use in real-time tracking of viral replication and pathogenesis. These replication-competent reporter-expressing IAVs also provide an efficient tool for high-throughput screening (HTS) of novel anti-IAV therapeutics. We also discuss the advantages and limitations of current fluorescent and luciferase reporter-expressing IAVs to track viral infections in cultured cells and/or animal models, and provide perspectives on future directions in this rapidly advancing field.

## INTRODUCTION

Influenza viruses are enveloped single-stranded negative-sense viruses that belong to the family *Orthomyxoviridae*. Influenza viruses are primarily classified into four genera, namely Alphainfluenzavirus, Betainfluenzavirus, Gammainfluenzavirus, and Deltainfluenzavirus, that include four corresponding influenza species: A (IAV), B (IBV), C (ICV), and D (IDV), respectively (1). IAVs and IBVs are the primary etiologic agents of seasonal influenza epidemics in humans responsible for significant public health and economic concerns (1). In addition, IAVs have been responsible for annual epidemics and occasionally pandemics of profound consequences to humans (1). The 20th century witnessed three influenza pandemics caused by IAVs: the Spanish flu (H1N1) in 1918, the Asian flu (H2N2) in 1957, and the Hong Kong flu (H3N2) in 1968. In 2009, a quadruple-reassortant swine-origin H1N1 IAV emerged and caused the first influenza pandemic of the 21st century (2, 3).

In parallel, avian influenza viruses, including highly pathogenic avian influenza (HPAI) H5Nx (x = N1, N5, N6, and N8) viruses, have caused millions of poultry losses in Africa, Asia, Europe, and North America (4). Importantly, HPAI H5Nx viruses could cross the species barrier to infect several mammalian species, including humans (1, 5). Since the entry into the United States of America in 2021, HPAI H5N1 (clade 2.3.4.4b) viruses have expanded their host range to include other farm animals, such as cattle, and are responsible for infecting other mammal species, including cats and humans, as well as birds (5, 6). To date, 71 human cases have been detected positive for the HPAI H5Nx viruses, including two fatalities (5, 7).

**Fig 1:**
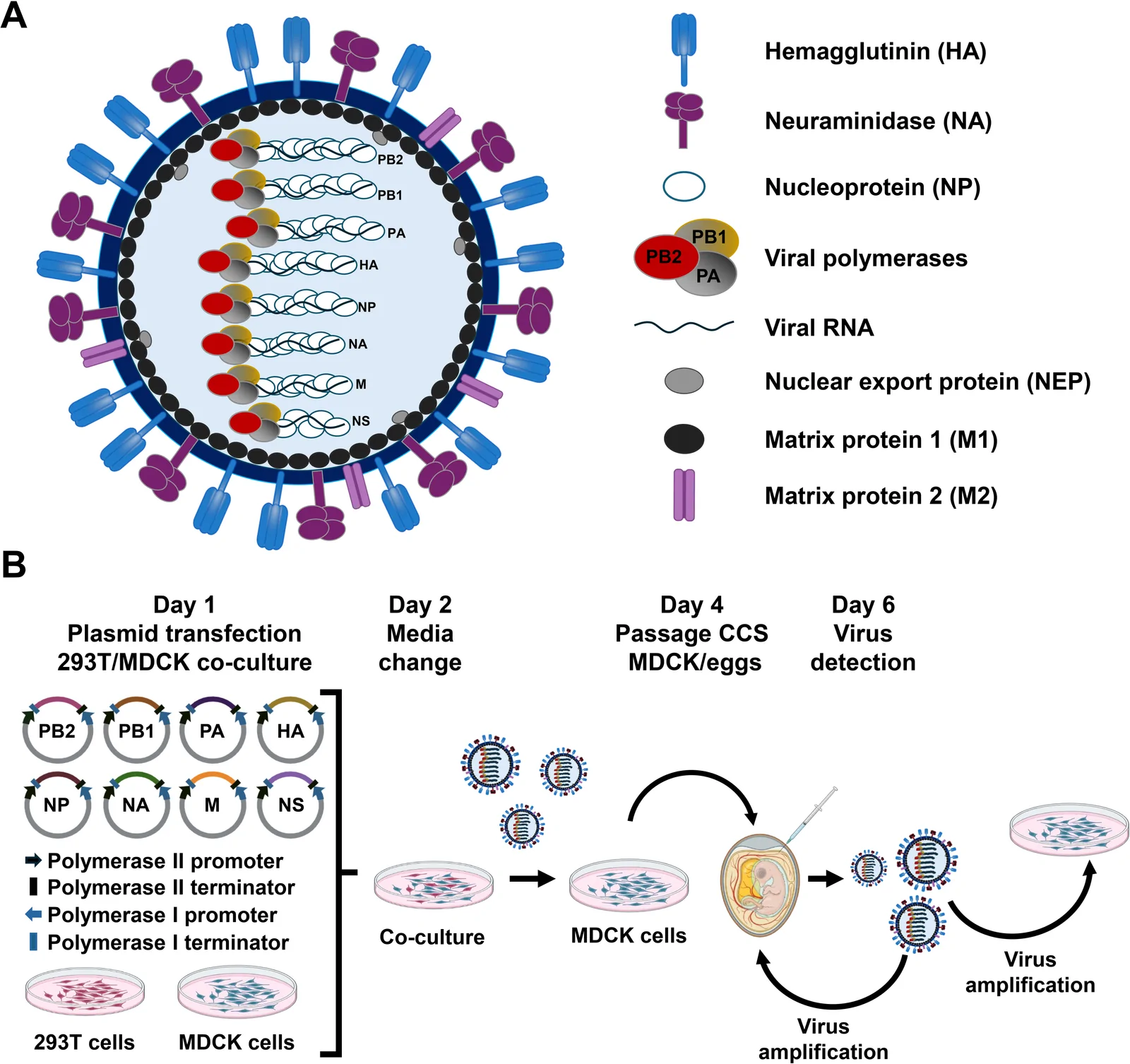
IAV structure and reverse genetics (RG) approaches to generate recombinant IAV. (**A**) Genome organization and virion structure of IAV: IAVs are single-stranded, negative-sense RNA viruses with a segmented genome consisting of eight vRNA segments (PB2, PB1, PA, HA, NP, NA, M, and NS). The virion envelope is made of a lipid bilayer containing the surface glycoproteins HA and NA, and M2. HA mediates viral attachment to sialic acid-containing receptors on host cells, whereas NA facilitates the release of newly formed virions from infected cells. Beneath the viral envelope lies the M1 protein, which plays a critical role in virion assembly and budding. vRNPs, composed of vRNA encapsidated by NP and associated with the polymerase subunits PB2, PB1, and PA, mediate viral genome replication and gene transcription. IAV NEP is responsible for the nuclear export of vRNPs during virus replication. (**B**) RG approaches to generate recombinant IAVs expressing reporter genes: The eight IAV viral segments are cloned into bidirectional ambisense plasmids containing RNA polymerase I (Pol I) and polymerase II (Pol II) promoter–terminator elements that drive vRNA (Pol I) and protein (Pol II) expression in opposite orientations. Co-cultures of human 293T and MDCK cells are co-transfected with equal amounts of the eight ambisense plasmids using a suitable transfection reagent. After 24 h, the transfection medium is replaced with infection medium. At 48 h, cell culture supernatants (CCSs) are collected and used to infect fresh monolayers of MDCK cells or chicken embryonated eggs. Cytopathic effect (CPE) is monitored daily in MDCK cells, and CCSs and allantoic fluid (chicken embryonated eggs) are collected to assess virus rescue by hemagglutination (HA) assay. Once the presence of the recombinant IAV expressing reporter genes is detected by HA assay, fluorescent microscopy (for fluorescent viruses) or plate readers (for bioluminescent viruses) are used to assess reporter gene expression. Recombinant IAVs expressing reporter genes can be amplified by passage in MDCK cells or chicken embryonated eggs to generate viral stocks. The figure has been created using Biorender.com.

IAV has eight negative sense, single-stranded RNA segments, including PB2 (polymerase basic 2), PB1 (polymerase basic 1), PA (polymerase acidic), HA (hemagglutinin), NP (nucleoprotein), NA (neuraminidase), M (which encodes M1 and M2 proteins by a spliced mRNA product), and NS (which encodes NS1 and NEP by a spliced mRNA product) (Fig. 1A) (1). The PB2, PB1, and PA segments encode the three polymerase subunits, where PB2 is involved in mRNA cap recognition, PB1 in RNA elongation, and PA in endonuclease activity to initiate the cap-snatching process (1). HA is a glycoprotein that is involved in receptor binding and fusion activities (8). NP is the RNA-binding protein involved in nuclear import regulation and viral RNA protection (1). NA is the second major viral glycoprotein, with sialidase activity and responsible for virus release (1). IAVs are classified into different subtypes based on the antigenic surface glycoproteins HA (19 subtypes) and NA (11 subtypes) (1, 9). The M segment encodes the major matrix 1 (M1) protein, which interacts with the viral ribonucleoprotein (vRNP) complexes, regulates RNA nuclear export, and contributes to viral assembling and budding (1). Matrix 2 (M2), an ion channel protein, is expressed alongside M1 through an alternative RNA splicing mechanism and engages in virus uncoating and assembly (1). The smallest viral segment, NS, encodes the non-structural 1 (NS1) protein, an interferon (IFN)-antagonistic protein that regulates host gene expression (10). Following RNA splicing, the NS also encodes the nuclear export protein (NEP), which mediates the nuclear export of viral (v)RNAs to the cytoplasm of infected cells (1).

IAV vRNPs comprise a vRNA segment coated with multiple NP molecules and associated with a single heterotrimeric RNA-dependent RNA polymerase (RdRp) complex (PB2, PB1, and PA), forming the minimal functional unit responsible for viral RNA genome replication (vRNA↔cRNA) and gene transcription (vRNA→mRNA) in the nucleus of infected cells (11). Elucidation of the composition and functions of the vRNP complex was a pivotal step that enabled the emergence of reverse genetics (RG) of influenza viruses, allowing precise genetic manipulation of viral genomes and the generation of recombinant viruses from plasmid-based systems (11, 12).

RG approaches are a powerful tool that utilize plasmid DNA to generate recombinant viruses, including IAV (13–16). These RG techniques allow the generation of recombinant viruses to understand the function of viral proteins or the impact of amino acid variations on the function of viral proteins and studying viral pathogenicity (17–20). RG techniques have also been used for the development of attenuated viruses for their implementation as live attenuated vaccines, to generate viruses limited to a single cycle of viral replication, and as vaccine vectors by expressing foreign genes (15, 18, 21). Bidirectional plasmid-based RG systems of IAV are the most used approach to generate recombinant IAVs and involve the cloning of the eight vRNA segments as cDNA into bidirectional plasmids (Fig. 1B) (14, 22). Each plasmid contains expression cassettes, in opposite orientation, for RNA polymerase I (Pol I) and RNA polymerase II (Pol II), enabling the synthesis of vRNAs and viral proteins, respectively, from the same plasmid DNA (14, 22). The Pol I cassette is made of Pol I promoter and terminator sequences, while the Pol II cassette, which flanked the Pol I cassette, is made of Pol II promoter and terminator sequences (14, 22). Pol I is species-specific and therefore relies on the specific match of cell lines with the Pol I promoter used; whereas Pol II can be used in different cell lines (23). For the generation of reporter-expressing recombinant IAVs, reporter genes of interest are cloned into plasmids within a specific viral segment (24, 25). The eight IAV ambisense plasmids are typically transfected into a co-culture of human 293T cells, if using a human Pol I promoter, and canine MDCK cells, due to the high transfection efficiency of the 293T cells, and the feasibility of MDCK cells to support efficient IAV replication. In the case of recombinant IAVs expressing reporter genes, virus rescue and replication can be easily tracked by reporter expression using fluorescent microscopy or luciferase plate readers, in the case of recombinant IAVs expressing fluorescent and luciferase proteins, respectively (24, 25).

## IAV INFECTION AND VIRAL MODULATION OF HOST SPLICING

IAV initiates infection by binding to host cell surface receptors via the viral HA glycoprotein. The primary receptor is N-acetylneuraminic acid (sialic acid), with α2,6 linkages predominant in human respiratory epithelial cells or α2,3 linkages dominant in avian species (26, 27). In addition to sialic acids, IAV can interact with secondary attachment factors, including MHC class II molecules on immune cells, C-type lectins, such as DC-SIGN, heparan sulfate proteoglycans, and gangliosides, which facilitate viral attachment and modulate tropism (28–31). During IAV viral replication, HA is cleaved into HA1 and HA2 by serine proteases (32). This post-translational modification is central for IAV infectivity (32). The HA2 subunit mediates fusion of the virus envelope and cell membranes, whereas the HA1 has the receptor binding and antigenic sites (32). After the attachment of IAV HA protein to the sialic acid-containing receptors, the virus is endocytosed (32). A low pH causes a conformational change in HA and the endosome membrane (32). Briefly, the pH of the endosome becomes lower when hydrogen ions are pumped into the virus particle through the activity of the M2 ion channel protein (33). Consequently, protein–protein interactions are disrupted due to the low pH (pH = 5–6), allowing the vRNPs to be released into the host cell cytoplasm (33). Once released, the vRNPs are directed into the host cell nucleus along with the viral polymerase PB2, PB1, and PA proteins. Viral RNA synthesis occurs in the nucleus, where viral mRNAs are transcribed from the vRNAs and serve as templates for viral protein translation. The vRNA also serves as templates for viral replication, resulting in the synthesis of complementary positive-sense cRNAs that are replicated to provide new vRNAs for nascent virions (34). The viral RdRp complexes part of the vRNPs are responsible for vRNA gene transcription to synthesize mRNA and vRNA genome replication to generate cRNAs that are used as templates to generate more vRNAs (35).

The poly(A) tail of IAV mRNA is encoded in negative-sense vRNA as a stretch of five to seven uracil residues, which the viral RdRp transcribes into the positive sense as a string of adenosines that form the poly(A) tail (36). The mRNA capping occurs in a similarly unique manner, in which the PA and PB2 proteins cleave and steal the 5′-capped primers from host pre-mRNA transcripts to initiate viral mRNA transcription; a process called “cap snatching” (36). Once polyadenylated and capped, the viral mRNAs can be exported from the nucleus and translated in the cell cytoplasm similarly to host mRNAs (37). Meanwhile, the M1 protein interacts with NEP and mediates the nuclear export of newly vRNAs into the cytoplasm (37). HA, NA, and M2 are synthesized from the viral mRNA by the ribosomes present on the endoplasmic reticulum (37). They are folded and imported into the Golgi complex, where they are directed to the cell membrane after undergoing post-translational modifications (37). M1 protein accumulation occurs in the cytoplasm near the cell membrane, initiating virus budding at the cell membrane (37). IAV HA proteins are released from bound sialic acid receptors by the sialidase activity of IAV NA protein, resulting in virus release from infected cells (32, 37).

Splicing is a fundamental process in eukaryotic gene expression. During this process, introns (noncoding regions) are removed from precursor mRNA (pre-mRNA), allowing exons, the true coding sequences, to be precisely joined together to form mature mRNA suitable for translation (38, 39). In addition, pre-mRNAs can undergo alternative splicing, generating multiple mRNA variants from a single primary transcript and thereby expanding protein diversity (39). In eukaryotic cells, splicing is carried out by the spliceosome, a large ribonucleoprotein (RNP) complex composed of five small nuclear RNAs, namely U1, U2, U4, U5, and U6, along with numerous regulatory proteins (38). The accurate function of the spliceosome depends on specific *cis-*acting elements within the pre-mRNA that are recognized by this complex, including the 5′ splice site donor (SD), the branch site, and the polypyrimidine tract (Y)n upstream from the conserved AG dinucleotide at the 3′ splice site acceptor (SA) (38, 40).

**Fig 2:**
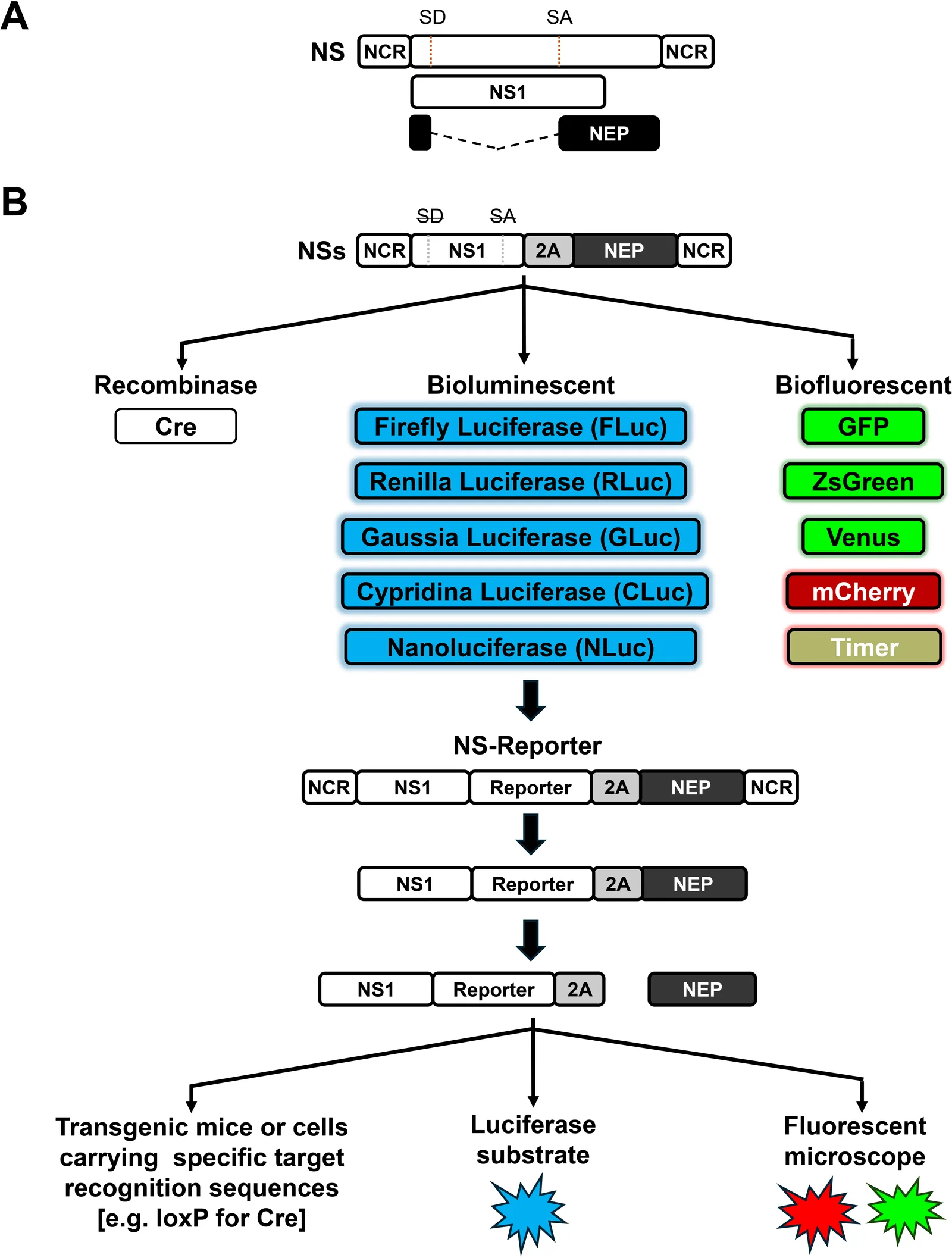
Schematic representation of parental and modified IAV NS segments for expression of reporter genes. (**A**) Schematic representation of influenza WT NS segment: IAV NS viral segment encoding NS1 (white) and NEP (black), flanked by non-coding regions (NCR). Spliceosome recognition depends on key *cis*-acting elements in the viral pre-mRNA, including the 5′ splice donor site (SD), the branch point, and the polypyrimidine tract (Y)n located upstream of the conserved AG dinucleotide at the 3′ splice acceptor site (SA). (**B**) Schematic representation of the modified NS segment for the expression of foreign genes: diagram of IAV NS segment encoding either recombinases (e.g., Cre), luciferases (e.g., FLuc, RLuc, GLuc, CLuc, or NLuc), or fluorescent proteins (e.g., green fluorescent protein [GFP], ZsGreen, Venus, mCherry, or Timer) reporter genes. The reporter gene is fused to the C-terminal of NS1. NS1 and NEP are produced from a single transcript using the porcine teschovirus-1 (PTV-1) 2A self-cleaving peptide (gray). To prevent RNA splicing of the viral NS constructs, silent mutations are introduced at the SD and SA sites (indicated by strikethrough). Following infection, recombinases mediate site-specific recombination at loxP sites, resulting in permanent activation of a reporter gene (e.g., tdTomato) in infected mice or cells carrying specific target recognition sequences. Luciferase-expressing viruses are detected by bioluminescence. Fluorescence-expressing viruses are monitored by fluorescence microscopy. The figure has been created using Biorender.com.

Splicing of IAV mRNAs was first recognized in the late 1970s following the identification of an additional 11-kDa viral polypeptide, NS2, for non-structural 2 protein (currently known as NEP), in infected cells (41). The NS segment produces multiple transcripts through alternative splicing. NS1 is encoded by unspliced colinear mRNA transcripts, while NEP is shorter and is generated from the spliced mRNA transcript (Fig. 2A). Its 5′ portion (10 amino acids) is identical to the NS1 transcript, while the second portion corresponds to the terminal region of the NS segment (Fig. 2A). The SD and SA dinucleotides (GU and AG) and their surrounding sequences are highly conserved and closely resemble human consensus splice sites (Fig. 2A), supporting efficient recognition by the host splicing machinery. In addition to NEP, a third NS-derived transcript in a mouse-adapted human IAV (A/Hong Kong/01/1968 H3N2) was reported (42). This transcript encodes NS3 and contains a novel 5′ splice site (GG/GUA) while retaining the same 3′ splice site used for NEP mRNA (38, 42). However, this alternative splicing event has been observed only in a limited number of IAVs, and it is suggested to arise following virus adaptation to the host, potentially involving the emergence of new splice sites. Such events may enable the production of novel viral proteins that enhance IAV replication efficiency (38, 42).

The levels of mRNAs and proteins derived from spliced viral transcripts change dynamically during IAV infection. NS1 protein is detected early after infection, followed by the appearance of M1, and later by the expression of NEP and then M2 (43). Although the proportion of spliced to unspliced NS mRNAs is thought to remain relatively constant at around 10% throughout viral infection (44), NS1 protein continues to accumulate over time and reaches its highest levels during the later stages of IAV infection (44). Therefore, IAV NS1 protein is the most abundantly expressed protein during viral infection (Fig. 2A) (10). IAV NS1 plays a significant role in counteracting the antiviral IFN response and in regulating host gene expression during viral replication (17, 19). IAV NS1 interacts with multiple key regulators of IFN induction and signaling pathways, including retinoic acid-inducible gene I (RIG-I), tripartite motif containing 25 (TRIM25), protein kinase R (PKR), the protein activator of PKR (PACT), the regulatory subunit p85β of phosphatidylinositol 3-kinase (PI3K), and PDZ domain-containing proteins (10). Due to the ability of IAV NS1 to suppress the production of IFN in host cells, NS1 is a key determinant in viral replication and pathogenicity (10, 17, 19). IAV NS1 varies in length among different viral subtypes, which may influence its functional properties and interactions with host cellular factors (10). IAV NS1 is divided into two distinct alleles: A and B (10). On the other hand, the NS segment expresses NEP from a spliced mRNA to efficiently contribute to the nuclear export of vRNAs, viral budding, and viral replication and transcription (45).

The disruption or the rearrangement of the conserved splicing sites within IAV NS segment has been exploited to enable the insertion of reporter genes (24, 25). By modifying the normal splicing pattern, foreign sequences can be incorporated into the NS segment while preserving essential viral functions. This strategy enables the generation of recombinant IAVs expressing foreign genes like fluorescent, recombinase, and luciferase proteins for studying viral genome replication and transcription, and host–virus interactions (Fig. 2B) (24, 25). These recombinant IAVs expressing reporter genes have been shown to represent valid viral surrogates to easily assess and quantify viral infection *in vitro* without the need for secondary approaches (e.g., antibody-based assays) to confirm the presence of IAV in infected cells. They are also valuable tools for screening antiviral compounds and evaluating neutralizing antibodies (nAbs) that inhibit or block viral infections, respectively (24, 25). Moreover, reporter-expressing recombinant IAVs can be used in validated animal models to monitor viral dissemination *in vivo* in the entire animals, as well as *ex vivo* in tissues collected from infected animals using *in vivo* imaging systems (IVIS) (24, 25).

Unlike IAVs expressing recombinases, which require specialized transgenic reporter mouse models for signal detection (46–49), fluorescent- and bioluminescent-expressing IAVs can be used directly in conventional cell cultures and animal models to track viral infections. This makes them more versatile, broadly applicable, and easier to implement for studying IAV replication and transcription, tissue tropism, pathogenesis, and antiviral efficacy. Accordingly, the following sections focus on the development, characteristics, and applications of these reporter IAVs in influenza research.

## GENETIC ENGINEERING APPROACHES TO GENERATE REPORTER IAVs

The generation of reporter IAVs requires a delicate balance between robust reporter expression and preservation of viral fitness. The IAV genome is tightly constrained across its eight negative-sense RNA segments, with the potential of inserting foreign genetic material disrupting native packaging signals, protein–protein interactions, or replication/transcription processes. When evaluating strategies to construct replication-competent reporter-expressing IAVs, methodologies fall into three major categories: surface glycoprotein modifications, polymerase subunit fusions, and NS segment re-engineering (Table 1). Unlike non-structural protein-encoding genes, modifying structural genes often introduce severe functional bottlenecks. For instance, N-terminal signal peptide fusions on segment 4 (HA) or monocistronic terminal fusions on segment 6 (NA) can preserve near-wild-type (WT) viral replication kinetics, but they represent a risk in altering viral entry, budding, or surface antigenicity. Notably, N-terminal fusions to IAV NA can drop NA enzymatic activity to as low as 17% due to structural alterations of the protein stalk (50). Moreover, the C-terminal fusions with terminal packing sequence duplications on the polymerase subunits PA or PB2 have been developed as highly effective systems. When paired with small bioluminescent proteins like NLuc (~19 kDa), these IAVs exhibit near-native viral fitness and exceptional stability across multiple passages (51, 52). However, this approach is less effective for rescuing IAV containing larger reporter genes like fluorescent proteins, which often lead to reduced viral replication efficiency in comparison to WT virus (53). Likely, full coding-region replacements or substitutions of essential core genes, such as HA or PB1 (54, 55), allow stable reporter expression but eliminate autonomous viral replication, resulting in replication-defective IAVs that are limited to a single-cycle round of infection. As a result, their use is primarily confined to *in vitro* assays using specialized cell helper systems. Therefore, for applications that require uncompromised, multi-cycle replication competence, investigators frequently turn to the NS segment 8 splice-site knockout and a continuous polyprotein expression strategy (18, 24, 25, 48). This system is designed to bypass the naturally overlapping coding organization of segment 8. In WT IAVs, NS1 and NEP are produced through alternative splicing of a single transcript. By genetically engineered mutations of the alternative SA site, this splicing dependency is removed, allowing the segment to be converted into a single, continuous open reading frame (ORF). A self-cleaving 2A peptide, such as the one encoded by the porcine teschovirus-1 (PTV-1), is then utilized to drive the expression of both vital viral proteins and the reporter from a single mRNA transcript (NS1-Reporter-2A-NEP).

**TABLE 1:** Genetic approaches for the development of reporter-expressing IAVs^*a*^

Modified segment	Virus backbone	Reporter gene	Design strategy	Reporter stability/ attenuation	Replication competence	Primary application	Ref.
Segment 1 (PB2)	PR8	GLuc	The reporter gene is fused to the C-terminus of the polymerase subunit or linked by a 2A peptide; the terminal packaging signals of the modified segment are synthetically duplicated downstream of the reporter to preserve genomic packaging efficacy	Stable; ~10-fold decrease *in vitro*; ∼50- to 100-fold decrease *in vivo*	Competent	*In vitro* *In vivo*	(56)
	WSN	Split-GFP11 (GFP1-10 expressed in cells)		Stable; not attenuated	Competent	*In vitro*	(53, 57)
	H7N7	GLuc		Stable; substantial attenuation *in vivo*	Competent	*In vitro*	(48)
	H7N7	GFP		Stable; not attenuated	Competent	*In vitro*	(48)
	PR8	Cre		Stable; not attenuated	Competent	*In vitro* *In vivo*	(49)
Segment 3 (PA)	WSN	NLuc	The reporter gene is fused to the C-terminus of the polymerase subunit or linked by a 2A peptide; the terminal packaging signals of the modified segment are synthetically duplicated downstream of the reporter to preserve genomic packaging efficacy	Stable; minimal attenuation	Competent	*In vitro* *In vivo*	(51)
	pH1N1	NLuc		Stable; minimal attenuation	Competent	*In vitro* *In vivo*	(58)
	WSN	Seven tandem GFP11 repeats (GFP1-10 expressed in cells)		Stable; ~100-fold decrease *in vitro*	Competent	*In vitro*	(59)
Segment 4 (HA); Segment 8 (NS)	PR8	NLuc (HA); Venus mCherry (NS)	NLuc is fused in the N-terminus of the HA via a 2A peptide; Venus or mCherry is linked to the C-terminus of NS1 via a 2A peptide	Stable; highly attenuated *in vivo*	Competent	*In vitro* *In vivo*	(60, 61)
Segment 6 (NA)	PR8	GFP	GFP is fused to either the N-terminus (rPR8-eGFP+NA) or the C-terminus (rPR8-NA+eGFP) of NA via a 2A peptide	Stable; rPR8-eGFP+NA attenuated; rPR8-NA+eGFP minimal attenuation	Competent	*In vitro* *In vivo*	(50)
	PR8	GLuc	GLuc is linked to C-terminus of NA via a 2A peptide	Stable; ~100-fold decrease *in vitro*	Competent	*In vitro* *In vivo*	(62)
Segment 8 (NS)	PR8	GFP	Genome rearrangement to remove NS splicing machinery, and the reporter gene is fused to the C-terminus of NS1 and linked to NEP via 2A peptide	Stable; ~100-fold decrease *in vitro*	Competent	*In vitro* *In vivo*	(63)
	PR8	Timer		Stable; not attenuated	Competent	*In vitro* *In vivo*	(64)
	pH1N1						
	PR8	NLuc		Stable; not attenuated	Competent	*In vitro* *In vivo*	(24, 25)
	pH1N1						
	H5N1						
	PR8	Venus GFP mCherry		Stable; passaged to make mouse adapted	Competent	*In vitro* *In vivo*	(65)
	H5N1	Venus mCherry		ND	Competent	*In vitro*	(66)
	H7N7	GFP Azurite dsRed Cre	Reporter genes are flanked by two genetically distinct PTV-1 2A-encoding sequences (NS1-2A-reporter-2A-NEP)	Stable; not attenuated	Competent	*In vitro* *In vivo*	(48)
		RLuc GLuc		Partially attenuated but highly stable in cell culture	Competent	*In vitro*	(48)
	PR8	mCherry NLuc	The reporter gene is cloned instead of the viral NS1 protein	Stable; slightly attenuated	Conditional replication competent	*In vitro* *In vivo*	(67)
	PR8	GFP	The reporter gene is fused to the C-terminus of the NS1 via a peptide sequence comprising a caspase recognition site that was inserted upstream of the GFP protein	Stable; not attenuated	Competent	*In vitro* *In vivo*	(68)
	PR8	GFP	The reporter is expressed using a bicistronic expression strategy based on the insertion of an overlapping UAAUG stop-start codon cassette into the NS gene	Stable; not attenuated	Competent	*In vitro* *In vivo*	(69)

^*a*^ PR8, influenza A/Puerto Rico/8/1934 H1N1; WSN, influenza A/WSN/1933 H1N1; H7N7, influenza A/SC35M H7N7; pH1N1, influenza A/California/04/2009 pandemic H1N1; H5N1, influenza A/Texas/37/2024 H5N1; GLuc, Gaussia luciferase; NLuc, Nanoluciferase; Cre, cyclization recombinase; RLuc, Renilla luciferase.

While early iterations of this strategy utilizing large fluorescent proteins, such as the green fluorescent protein (GFP), suffered from a 1–2 log reduction in viral replication (63, 70), the paradigm has shifted with the integration of smaller reporter fluorescent or luciferase proteins (24, 60, 67, 71, 72). The attenuation observed in fluorescent-expressing IAV using PTV-1 2A linkers may be partly attributable to reduced NEP expression (70). This decrease was associated with a two-log reduction in progeny virus production despite normal expression of structural proteins like NP, indicating impaired viral replication (70). When this NS-engineered design is combined with small bioluminescent reporters like NLuc, the structural burden or the impact of the inserted foreign gene on the virion is minimized, preserving near-native viral fitness (24, 25). Thus, these constructs avoid the severe attenuation associated with the rearrangement of the NS segment while maintaining the ability to monitor viral replication, dissemination, and longitudinal *in vivo* imaging. The ability to circumvent splicing constraints, genetic stability across passages, and impact on native replication, the NS-based polyprotein strategy emerges as the premier approach for generating replication-competent reporter-expressing IAVs similar to those of their WT counterparts.

## RECOMBINANT IAVs EXPRESSING REPORTER GENES FROM THE NS SEGMENT

**Fig 3:**
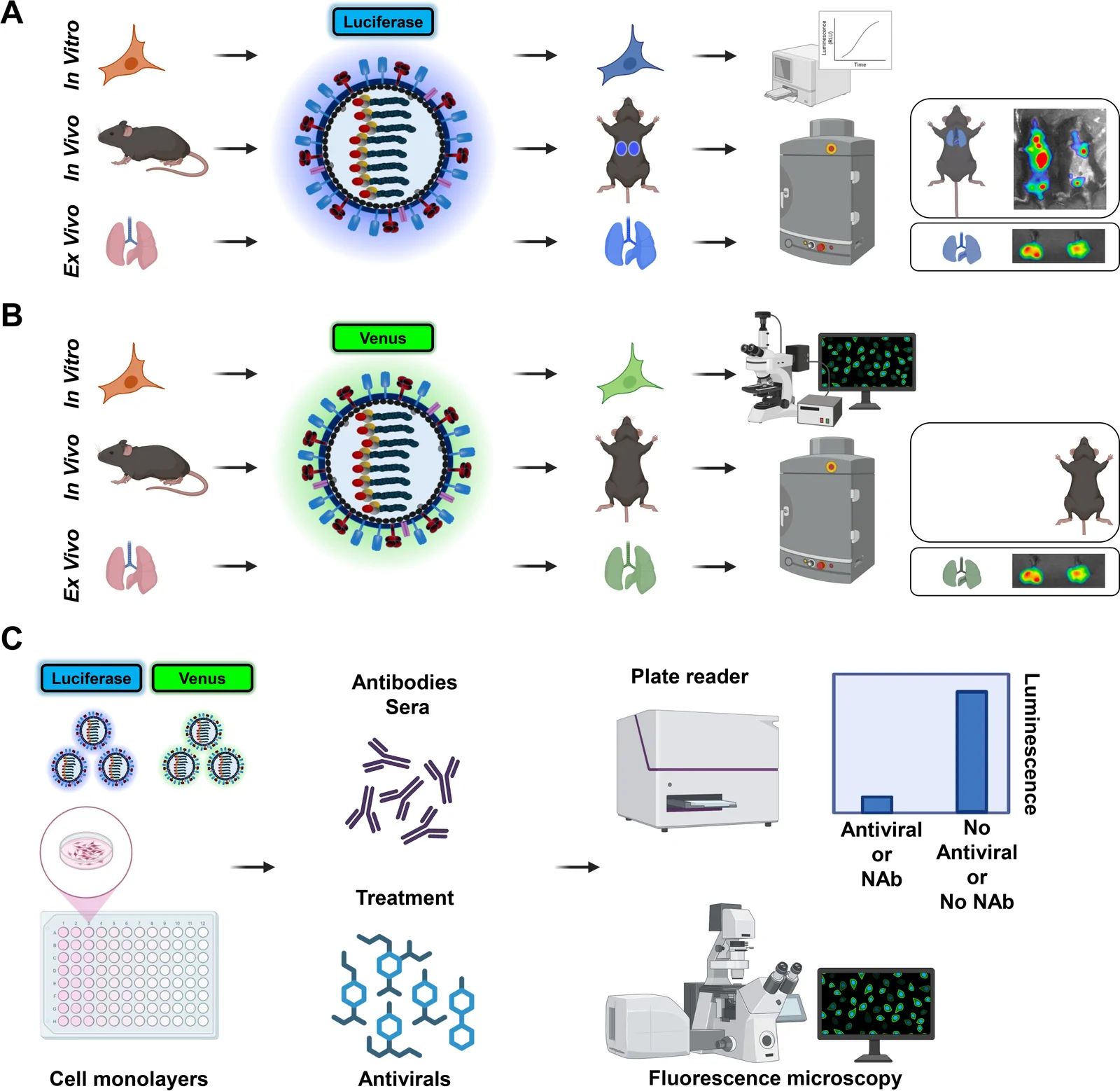
Uses of recombinant IAVs expressing bioluminescence or fluorescent reporter genes. (**A**) Recombinant IAVs expressing luciferase: Recombinant IAVs expressing luciferase (e.g., NLuc) reporter genes enable sensitive quantification of viral replication *in vitro* by measuring luciferase activity in CCSs, although intracellular viral replication cannot be directly visualized and is instead determined by CPE or luciferase assays. *In vivo*, NLuc-expressing recombinant IAVs allow noninvasive longitudinal monitoring of viral replication in small animal models using IVIS, thereby reducing the number of animals required for a study. Recombinant IAVs expressing NLuc also allow *ex vivo* assessment of viral replication in the tissues of infected animals. However, *in vivo* or *ex vivo* detection of NLuc in animals infected with recombinant IAVs requires administration of the NLuc substrate. For NLuc-expressing recombinant IAVs, viral replication is monitored longitudinally in live animals using IVIS. On imaging days, mice are ventrally shaved to expose the thoracic and abdominal regions, anesthetized, and administered with the NLuc substrate via retro-orbital or intraperitoneal injection. NLuc signal is acquired immediately in the ventral position using an IVIS. Depending on the experimental design and viral strain, imaging may be run on alternate days to monitor infection kinetics. *Ex vivo* imaging can also be performed by excising organs (e.g., lungs) to assess tissue-specific viral distribution. (**B**) Recombinant IAVs expressing fluorescent proteins: recombinant IAVs expressing fluorescent reporter genes (e.g., Venus) allow direct visualization of viral infection and replication dynamics in infected cells *in vitro* by fluorescence microscopy, facilitating studies at the cellular level. In contrast to luciferase reporters, fluorescent-expressing reporter IAVs cannot be readily used to monitor viral infection using whole-body live imaging and are typically assessed *ex vivo* in tissues collected from infected animals. For fluorescent-expressing recombinant IAVs, viral infection is evaluated *ex vivo* by harvesting tissues (e.g., lungs) at defined times post-infection and visualizing fluorescence expressing *ex vivo* using IVIS. (**C**) Reporter-based *in vitro* assays for the identification and characterization of antivirals and nAbs using fluorescent- or luciferase-expressing recombinant IAVs: cell monolayers are seeded in 96-well plates and infected with either luciferase- or fluorescent-expressing recombinant IAVs. Following viral adsorption, the virus inoculum is removed, and cells are incubated with post-infection media in the presence of serial dilutions of antibodies or sera, or antiviral compounds. For luciferase-expressing recombinant IAVs (e.g., NLuc), neutralizing or antiviral activities are determined by measuring luciferase activity in CCSs using a luciferase plate reader. For fluorescent-expressing recombinant IAVs (e.g., Venus), neutralizing or antiviral activities are monitored by fluorescent microscopy or by using a fluorescent plate reader. The figure has been created using Biorender.com.

IAVs expressing both fluorescent and luciferase reporter proteins from different viral segments have been described in the literature (48, 51, 61, 63, 73). However, recombinant IAVs engineered to express reporter genes from the NS segment have proven particularly efficient in tracking viral replication *in vitro*, *ex vivo*, and *in vivo* (Fig. 3A and B) (24, 25). As the IAV NS segment is the smallest in size, it provides greater flexibility to accommodate foreign gene insertions while maintaining viral viability (24, 72). Furthermore, when a reporter gene is inserted into the IAV NS segment, the packaging signals are not disrupted (72). Additionally, given the strong expression of NS1 during viral infection, reporter genes engineered into the NS segment are expressed at higher levels (24, 25).

During IAV infection, the NS viral segment normally produces two proteins, NS1 and NEP, through alternative splicing of a single primary transcript (10). In the modified strategy described in Fig. 2B, this natural splicing dependency is eliminated by engineering the NS segment with altered SD and SA sites to express both NS1 and NEP from a single, non-overlapping transcript (10, 74–76). This is achieved by inserting 2A autoproteolytic cleavage sequences, like PTV-1, between the NS1 and NEP coding regions (77). The PTV-1 2A sequence is a short peptide motif that mediates a ribosome “skipping,” known also as “stop-carry on,” “Stop-Carry On,” or “StopGo” translation (78). During translation, the ribosome fails to form a peptide bond at the glycine-proline junction within the 2A motif, resulting in co-translational cleavage of the growing polypeptide chain. Consequently, two independent proteins are produced from a single ORF: the upstream NS1 protein that retains the majority of the 2A sequence at its C-terminus, and the downstream NEP as a separate product starting with a proline residue. This strategy ensures stoichiometric co-expression of NS1 and NEP without reliance on host splicing machinery, thereby preventing alterations in splicing efficiency that could influence viral replication or reporter gene stability. Moreover, the elimination of overlapping coding regions simplifies genetic manipulation of the NS segment and facilitates the insertion of heterologous sequences (e.g., reporter genes) while preserving essential NS1 and NEP functions. As a result, NS1 and NEP are expressed as two distinct proteins from a single transcript, improving genetic stability and expanding the versatility of engineered IAVs. Besides PTV-1 2A, other 2A sequences like Foot and Mouth Disease Virus 2A cleavage sequence have also been used to stably express a heterologous protein from recombinant IAVs (79).

## FLUORESCENT AND LUMINESCENT REPORTER-EXPRESSING RECOMBINANT IAVs

Reporter-expressing IAVs provide the advantage of studying viral replication without the need for secondary methodologies (24, 25). Reporter genes encoding fluorescent or luciferase proteins enable straightforward tracking and quantification of viral infections *in vitro*, *ex vivo*, or *in vivo* (24, 25). Reporter-expressing IAVs also provide the advantage of detecting infected cells directly by fluorescence or enzymatic activity, and, therefore, can be used in high-throughput screening (HTS) settings to facilitate the identification of nAbs or antivirals (Fig. 3C) (24, 25).

Recombinant IAVs containing a modified NS segment were first described in 2004, when the GFP ORF was successfully inserted into the NS segment (68). This GFP-expressing virus enabled direct visualization of infected cells by fluorescence microscopy, demonstrating the feasibility of using the NS segment to accommodate reporter genes (68). Since that initial report, numerous fluorescent reporter-based technologies with diverse properties and applications have been developed. In contrast to luciferase reporter proteins, fluorescent protein detection does not require substrate administration, thereby simplifying experimental workflows and enabling truly noninvasive longitudinal imaging (80). These systems can be readily integrated with other imaging modalities to achieve multicolor visualization of multiple biological parameters (80). In addition to GFP, commonly used fluorescent reporter genes to generate fluorescent-expressing replication-competent IAVs include the red fluorescent protein (RFP), Venus, monomeric (m)Cherry, or ZsGreen (81, 82).

For *in vivo* imaging applications, bioluminescent reporter genes (e.g., luciferases) enable noninvasive assessment of biological processes in living organisms (51, 71, 83). These reporters produce light through enzyme-substrate reactions without the need for external excitation, resulting in high detection sensitivity and minimal background signal (80). Luciferase-expressing viruses typically utilize Firefly luciferase (FLuc; size = 61 kDa; emission wavelength = 560 nm), Renilla luciferase (RLuc; size = 36 kDa; emission wavelength = 480 nm), Gaussia luciferase (GLuc; size = 36 kDa; emission wavelength = 470 nm), or Nanoluciferase (NLuc; size = 19 kDa; emission wavelength = 460 nm) (16, 18, 24, 25, 48, 51, 61, 63, 72, 73, 84). Fluorescent proteins absorb light at specific wavelengths and emit light at longer wavelengths, while luciferase reporters generate bioluminescence through enzyme-catalyzed reactions (84, 85).

The bioluminescent luciferase approaches have traditionally relied on FLuc (ATP-dependent and uses D-luciferin as a substrate) and RLuc (ATP-independent and uses Coelenterazine as a substrate); however, their use is often constrained by limitations in size, stability, and luminescence output (84). Among available alternative luciferases, NLuc is particularly well suited for deep tissue imaging due to its small size, exceptionally strong signal intensity, low background, and higher sensitivity compared with other luciferases (80). NLuc uses furimazine or fluorofurimazine as substrates (86) and offers several advantages over conventional luciferases, including greater stability, a smaller molecular size, and more than a 150-fold increase in luminescent intensity (84). Moreover, its substrate exhibits enhanced stability and reduced background signal, creating new opportunities for bioluminescence imaging (84, 86). NLuc is highly versatile and suitable for a broad range of *in vivo* and *ex vivo* applications (84).

Despite these advantages, NLuc also has certain limitations, such as suboptimal emission properties for some *in vivo* applications and reliance on unique substrates, which may restrict its use in specific research. Nevertheless, as technology continues to evolve, NLuc is likely to play a vital role in both preclinical and clinical research, with potential applications in disease detection, molecular imaging, and prophylactic and/or therapeutic monitoring. In the context of IAV, luciferase-based systems have been widely applied for rapid and quantitative assessment of viral replication, identification of antivirals or nAbs, and real-time monitoring of viral infection *in vitro*, *ex vivo*, and/or *in vivo*. These reporters enable sensitive detection of low levels of IAV and facilitate longitudinal analysis of infection dynamics without the need for repeated sampling from multiple animals.

Few studies have shown that NLuc-expressing IAVs are slightly attenuated compared to their WT counterparts (24, 25, 52). Nevertheless, viral fitness could be restored following successive virus passaging while maintaining reporter protein expression (52) or by incorporating compensatory mutations during initial rescue of recombinant IAVs to expand the tolerance of the IAV genome (83). For instance, Cai et al. employed directed evolution to generate a genetically stable NLuc-expressing influenza A/California/04/2009 pandemic H1N1 (pH1N1) IAV with restored WT-like replication and virulence. The parental pH1N1 IAV containing NLuc inserted into the PA segment exhibited attenuation, reduced replication kinetics, and lower virulence *in vitro* and *in vivo* (52). Following 12 serial passages in DBA/2 mice, the adapted virus displayed enhanced NLuc expression, improved fitness, and restored pathogenicity comparable to the initial NLuc pH1N1 virus. Adaptive mutations in PB2 (E158G, C1161T, and C1977T) and PA (D479N) were able to enhance viral mRNA transcription and compensate for reporter-associated attenuation (52). Moreover, the mouse-adapted virus maintained stable NLuc expression while enabling sensitive noninvasive imaging of pH1N1 infection, supporting its application in studying virus–host interactions and evaluating prophylactic vaccine and therapeutic antiviral strategies.

Due to the limited packaging capacity of the IAV genome, trials have traditionally been limited to generating recombinant IAVs expressing either fluorescent or luciferase reporter genes. To address this limitation, Nogales et al. successfully generated a recombinant replication-competent bi-reporter IAV (BIRFLU) that stably expresses two reporter genes, one fluorescent and one luciferase, enabling the monitoring of IAV infection both *in vitro* and *in vivo* (61). In this reporter influenza A/Puerto Rico/08/1934 H1N1 (PR8), NLuc was incorporated into the HA segment, while the Venus or mCherry fluorescent proteins were cloned into the NS segment (60, 61). Shortly thereafter, the same group also reported the generation and characterization of two replication-competent NS1-deficient (ΔNS1) PR8 viruses in which NS1 was replaced with either mCherry or NLuc. The PR8 ΔNS1 mCherry virus retained the ability to replicate in cultured cells and in Signal Transducer and Activator of Transcription 1-deficient mice (67). Collectively, these studies contributed significantly to the development of advanced reporter-expressing IAV platforms for investigating viral replication, pathogenesis, and host–virus interactions in both cell culture and animal models.

## APPLICATIONS OF RECOMBINANT IAVs EXPRESSING REPORTER GENES

### *In vitro* assays to identify the presence of the virus in infected cells

Fluorescent-expressing IAVs are advantageous for studying virus replication *in vitro*, as infected cells can be readily visualized under a fluorescent microscope (61). A recombinant PR8 IAV expressing mCherry has been used to evaluate viral growth kinetics and to screen antiviral compounds and nAbs (81). This recombinant PR8 expressing mCherry can also be used to evaluate prophylactic vaccine efficacy and novel therapeutic antiviral strategies. Recombinant PR8 viruses expressing different fluorescent proteins (Color-flu) have been generated and adapted for use in mice (65). These multicolor viruses enable the study of viral co-infection within tissues and facilitate investigation of pathogenicity and viral interactions *in vivo* (65). Reporter-expressing recombinant IAVs have also been used to analyze viral reassortment by generating recombinant strains containing gene segments derived from human IAVs (72). Fluorescent-expressing recombinant IAVs have also been applied to study macrophage responses during IAV infection and to characterize host–virus interactions associated with HPAI viral strains (87). Moreover, a recombinant PR8 IAV expressing a fluorescent timer protein that undergoes a time-dependent shift in emission from green to red has been engineered to temporally resolve viral infection (64). This approach enables discrimination between primary and secondary infected cells and has been successfully employed to examine viral infection dynamics *in vivo* using mouse models. In addition, cell-to-cell spread and cell-free virion infection can be monitored in real time using live-cell video microscopy (88, 89).

### *Ex vivo and in vivo* real-time tracking of IAV replication

Recombinant IAVs expressing luciferase proteins also offer the possibility of tracking viral infection *in vivo* through live imaging systems, allowing monitoring of viral dissemination in the same animals at multiple times post-infection (Fig. 3A) (24, 25). This approach is particularly useful for evaluating antiviral therapeutics (24, 25). For example, a bioluminescent HPAI virus (A/Texas/37/2024 H5N1), derived from the first human case identified in Texas in 2024, was generated to track viral infections and to evaluate the antiviral activity of Baloxavir *in vivo* (24). This demonstrates the potential of reporter-expressing recombinant IAVs to facilitate rapid antiviral screening in preparation for future pandemics.

Recombinant IAVs expressing reporters can also be used to track viral infection in small animal models by *ex vivo* imaging (61). Following necropsy, lungs, or other organs where virus replicates can be imaged using IVIS. Combining *in vivo* and *ex vivo* imaging allows detailed analysis of viral tropism and facilitates therapeutic evaluation (90). This provides the advantage of observing viral infection in antigen-presenting cells collected from the tissues using flow cytometry (63). Intranasal inoculation with fluorescent IAVs has also shown expression in dendritic cells (DCs), macrophages, and neutrophils within 2 days post-infection (63). Importantly, fluorescent-expressing reporter IAVs are not limited to rodent models; they have also been used in zebrafish, which support IAV infection (91, 92). The swim bladder of zebrafish provides a physiologically relevant model analogous to human endothelial tissue (92). Multiplexed studies can be performed using IAV strains expressing different fluorescent proteins, enabling co-infection experiments to observe viral localization across multiple tissues (65).

## LIMITATIONS OF REPORTER-EXPRESSING RECOMBINANT IAV

Reporter IAVs differ in their optimal applications due to the intrinsic properties of fluorescent and luciferase reporter genes and detection methods. The selection of the right reporter gene to include in recombinant IAVs is important and also depends on the type of study and the type of reporter assay being conducted. For example, fluorescent reporter genes are more advantageous for *in vitro* studies to evaluate the presence of the virus in infected cells, whereas luciferase reporter genes are more advantageous to interrogate large libraries of antivirals in HTS settings *in vitro* and for *in vivo* studies due to their very low background and high signal-to-noise ratios (16). This is because fluorescent-expressing recombinant IAVs cannot be easily detected by live imaging in entire animals, while luciferase-expressing recombinant IAVs can be sensitively detected in entire animals using IVIS (24, 25). Fluorescent IAVs also have other limitations: excitation light is scattered above the plane of the target tissue, reducing signal intensity (61), and tissue autofluorescence generates background noise during imaging and poor tissue penetration, which reduce sensitivity (89). Typically, green fluorescent proteins (e.g., GFP) produce a stronger signal than red fluorescent proteins (e.g., RFP); however, tissue autofluorescence is generally higher in the green spectrum, which can reduce the effective signal-to-noise ratio (93). In addition, although GFP was successfully incorporated into the viral genome, the reporter gene was not stably maintained after four successive passages in MDCK cells, as confirmed by repeated plaque purification to isolate GFP-expressing virus populations (48, 63). In mice infected with GFP PR8 IAV, nearly 15% and 30% of plaques in lung homogenates were GFP negative by days 2 and 6 post-infection, respectively (63). This further confirms the limited genetic stability of fluorescent reporter-expressing IAVs both *in vitro* and *in vivo*. To address this limitation in the stability of recombinant IAV expressing GFP, a recent study generated replication-competent A/WSN/1933 (WSN) in which the GFP sequence was recoded using synonymous codons to mimic high NP-binding regions (H1N1/NS-rGFP), leading to the generation of a replication-competent virus with enhanced maintenance of GFP expression (94). Luciferase-expressing IAVs have been shown to be highly stable in MDCK cells and in mice compared to fluorescent-expressing recombinant IAVs (24, 25).

Although reporter IAVs have greatly facilitated HTS and real-time monitoring of viral infections, traditional virological assays, such as plaque assays, TCID_50_ (50% tissue culture infectious dose), and RT-qPCR, remain widely used for virus detection and quantification. Several studies have demonstrated a strong correlation between reporter signals and conventional readouts, supporting the use of reporter viruses as valid surrogates of viral infection (24, 25). However, reporter expression reflects transgene activity rather than direct measurement of infectious viral particles, and factors such as reporter stability and viral attenuation may influence the relationship between reporter signal and viral replication. Therefore, validation using traditional assays remains important, particularly when evaluating viral fitness, pathogenicity, antiviral or nAb efficacy, or vaccine-induced protection.

Issues with small animal *in vivo* imaging are mitigated by *ex vivo* imaging, making both fluorescent- and luciferase-expressing IAVs suitable for such analyses (61). However, luciferase-expressing IAVs, while optimal for *in vivo* and *ex vivo* imaging, cannot be used to identify the presence of the virus without exogenous administration of the luciferase substrate. Additional limitations of luciferase-based recombinant IAVs include the requirement for all components of the oxidation reaction to generate light (85), and the typical monochromatic emission of luciferase proteins, which restricts multiplexed labeling (80). Furthermore, for *in vivo* studies, luciferase substrates must be administered via invasive (injection) routes (24, 25), which can increase animal discomfort. Finally, the relatively high cost of luciferase substrates raises overall experimental expenses.

## CONCLUSIONS AND FUTURE DIRECTIONS

Various reporter-expressing, replicating-competent recombinant IAVs have been described in the literature. These viruses have become invaluable tools for studying multiple aspects of the biology of IAV. They have been used to investigate viral replication, protein functionality, efficacy of prophylactic vaccines, identification and characterization of therapeutic nAbs and antiviral drugs, and viral pathogenicity. The most significant advantage of reporter-expressing recombinant IAVs over their natural counterparts is the ability to minimize reliance on secondary methodologies to identify the presence of the virus *in vitro*, *ex vivo*, or *in vivo*, which can be expensive and time-consuming. Recombinant IAVs expressing reporter genes allow direct visualization of the virus in infected cells using fluorescent microscopy. These reporter-expressing recombinant IAVs also enable detecting the virus in infected small-animal models, such as mice, using *in vivo* or *ex vivo* imaging systems. This provides the advantage of identifying and characterizing prophylactics and/or therapeutics *in vitro*, including the interrogation of large libraries of compounds in HTS settings, and in *ex vivo* or *in vivo* experiments, minimizing costs and the number of animals used. These reporter-expressing recombinant IAVs also represent an excellent resource for pandemic preparedness by assessing nAbs and/or antivirals in cultured cells or small animal models. Recombinant IAVs expressing reporter genes can also be used to understand viral transmission and to facilitate identifying prophylactic vaccines. To date, fluorescent and luciferase proteins are the most used reporter genes to generate recombinant IAVs, and they both present advantages and disadvantages. Therefore, selecting the proper reporter gene to generate recombinant IAVs will depend on the type of study, where, in general, fluorescent proteins represent the best option for *in vitro* studies and luciferase proteins being the best option for *in vivo* and *ex vivo* studies, or for HTS *in vitro*. Despite their utility, reporter-expressing recombinant IAVs often show attenuation compared to natural isolates or recombinant WT counterparts. We and others have shown how recombinant IAVs expressing fluorescent proteins are attenuated compared to natural or WT IAVs (18, 24, 25, 61, 70). In contrast, luciferase-expressing recombinant IAVs are less attenuated compared to fluorescent-expressing recombinant IAVs, with similar levels of morbidity and mortality to those observed with natural or WT viral infections (24, 25). Overall, reporter-expressing recombinant IAVs provide a versatile and powerful platform for advancing influenza research, from basic virology to prophylactic vaccines and/or therapeutic antiviral development, and their continued refinement will enhance the study of viral dynamics, host–virus interactions, and the identification of new prophylactic and/or therapeutic interventions against these pathogens of concern to human health.
